# The Complexity of Aging: Managing Multimorbidity in Geriatrics

**DOI:** 10.7759/cureus.87615

**Published:** 2025-07-09

**Authors:** Sundas Qamar, Muhammad Sarfraz, Hifza Ishtiaq, Amna Akbar, Muhammad Iftikhar Khattak, Sohail K Raja, Marriam Khan

**Affiliations:** 1 Geriatrics, Russells Hall Hospital, Dudley, GBR; 2 Respiratory Medicine, Alexandra Hospital, Redditch, GBR; 3 Internal Medicine, Abbas Institute of Medical Sciences, Muzaffarabad, PAK; 4 Emergency and Accident Care, District Headquarter Hospital, Jhelum Valley, Muzaffarabad, PAK; 5 Research and Development, Celestial and Dimanche, Muzaffarabad, PAK; 6 Pulmonology, Abbas Institute of Medical Sciences, Muzaffarabad, PAK; 7 Internal Medicine, Indus Hospital and Health Network, Karachi, PAK

**Keywords:** chronic conditions, cognitive decline, elderly, geriatric care, healthcare access, healthcare utilization, health outcomes, multimorbidity, polypharmacy, socioeconomic status

## Abstract

This retrospective cohort study, conducted using electronic health records from a geriatric healthcare system in Pakistan, focused on multimorbidity and polypharmacy, involving 600 participants (297 females and 303 males) with a nearly equal gender distribution. The sample was diverse in terms of socioeconomic status, with 195 (32.5%) participants hailing from high socioeconomic backgrounds, 184 (30.7%) from low socioeconomic backgrounds, and 221 (36.8%) from middle-class backgrounds. Regarding education, 139 (23.2%) had postgraduate education, and 132 (22.0%) had no formal education. In terms of living conditions, 171 (28.5%) lived alone, 146 (24.3%) in assisted living, and 145 (24.2%) in nursing homes. The mean age of participants was 81.59 years [standard deviation (SD) = 10.3], and the majority exhibited moderate physical (42.7%) and cognitive decline (45.7%).

Most participants had moderate to severe multimorbidity (289 participants, 48.2%), and polypharmacy was common, with 321 (53.5%) taking five or more medications. Social isolation was high, with 289 (48.2%) participants experiencing difficulty engaging socially. Healthcare utilization was varied: 157 (26.2%) visited primary care once a month, and 169 (28.2%) attended specialist care twice a year. Hospitalization frequency averaged 4.51 visits (SD = 2.87), and emergency visits averaged 6.96 (SD = 4.22). Exploratory data analysis (EDA) identified significant correlations between polypharmacy and hospitalization frequency (r = 0.17, p<0.05) and a negative correlation with functional status (r = -0.11, p<0.05). Logistic regression revealed that polypharmacy [odds ratio (OR) = 1.35, p<0.05] and socioeconomic status (OR = 1.52, p<0.01) were significant predictors of adverse health events. This study underscores the complex healthcare needs of the elderly with multiple chronic conditions, highlighting the need for integrated care strategies to address both physical and mental health challenges.

## Introduction

Aging is a process that causes significant physical, cognitive, and social changes [1]. As life expectancy increases globally, the elderly population also increases, leading to an increased burden of health-related problems. One of the most prevalent areas of concern in geriatric care currently is multimorbidity, or the presence of multiple chronic diseases in one person [2]. Older adults are particularly susceptible to conditions including hypertension, diabetes, cardiovascular disease, osteoarthritis, dementia, and depression [3]. The complexity of multimorbidity can be overwhelming to manage because of the interaction between diseases, treatments, the decline in physiological capacity of older adults, and the greater potential of experiencing polypharmacy, which can produce a suboptimal quality of life [4]. According to a publication from the WHO, this group of 60 years and older, expected to exceed two billion people by 2050, will amount to more than 20% of the World’s total population [5]. In 2019, one in six people worldwide was aged 60 or older, and this number is on the rise. The Centers for Disease Control and Prevention (CDC) report that 85% of older patients (65+) have at least one chronic condition, with 60% having two or more conditions. Multimorbidity is now the norm, rather than the exception, for older populations worldwide [6].

Adopting a multimorbidity management approach in practice can be challenging due to the treatment and management pathways offered by healthcare, focusing on individual (isolated) diseases or conditions. However, some individuals experience multiple chronic conditions that, for older adults particularly, lead to increased complications and disability, more hospitalizations, and an increased risk of death [7]. Research has documented poorer health outcomes associated with multimorbidity when compared to having only one chronic condition. Further complicating management around these chronic conditions is the coexistence of mental health conditions, such as depression and decline in cognition, that are often associated with physical conditions. The National Institute on Aging reports that "among older adults, multimorbidity is associated with depression in approximately half of the cases" [8].

Research also demonstrates that multimorbidity leads to higher rates of healthcare resource utilization, including higher hospital admissions and emergency visits. Older adults with the highest burden of chronic conditions and/or multimorbidity have the highest burden of polypharmacy [9]. Reporting from the Irish longitudinal studies gives us shocking estimates; for example, as many as 40% of older adults take five or more medications, placing an extra burden on them and healthcare services [10]. The effects of polypharmacy include the risk of adverse drug interactions, poor medication adherence. In particular, multimorbidity is associated with both a reduction in functional status and those who have multiple conditions are three times more likely to lose independence than those with fewer health conditions [11].

The etiology of multimorbidity is multifactorial. Aging is associated with diminished immune function and diminished organ reserve; chronic diseases develop as a result of systemic inflammation; lifestyle factors such as poor diet and physical inactivity increasingly endanger our health; CDC states that 40% of older adults in the U.S. are obese, which contributes significantly to the development of diseases such as hypertension and diabetes; some environmental factors, like lack of access to healthcare, have led to increasing the burden of multimorbidity in old aged individuals [12]. Given the high number of elderly people living with multimorbidity, the healthcare community is grappling with the challenge of providing them optimal care and managing their health. Present healthcare systems do not fully take into account the complexity of multimorbidity, and healthcare can be fragmented, leading to inadequate outcomes for these people.

In light of this, we conducted a retrospective analysis of healthcare data among the geriatric population. It will assess multimorbidity and whether older adults experience any shortcomings and inadequacies in the treatments they receive. We aim to provide recommendations to facilitate transitions to integrated, patient-centered care [13]. We also seek to evaluate the prevalence, clinical outcome, management approaches, and care coordination for multimorbidity in geriatric individuals using archival databases. The objectives of the study are to examine the prevalence of multimorbidity in geriatric patients, understand the implications for healthcare utilization, assess treatment efficacy, and determine care gaps. We aim to offer recommendations on how to better manage multimorbidity among geriatric populations to improve their health outcomes.

## Materials and methods

Study design

This retrospective cohort study used data from 600 older adults (65 or older) extracted from the electronic health record system of a geriatric healthcare organization in Pakistan. The information provided extensive detail about demographic characteristics, clinical history, health behavior, comorbidity, medications prescribed, and healthcare outcomes. The analysis mainly focused on multimorbidity and its interaction with several key contributory factors, including functional and psychosocial status, healthcare utilization, and treatment outcomes (although, in a move away from the original aim, it also examined trends and relationships).

Data collection

Data were extracted from an electronic health record system maintained by a large provider of geriatric healthcare services in Pakistan. The original database provided records of patients aged 65 and older. Eligible patients were selected using a consecutive sampling approach over a specified period of time. Inclusion criteria consisted of patients aged 65 and older with documented diagnoses of at least two chronic conditions (e.g., hypertension, diabetes, cardiovascular disease, arthritis) and with medical records dating back to a minimum of one year. Patients were excluded if there were missing data on important variables such as demographics, comorbid conditions, medications, or health service utilization; if they were diagnosed with a terminal illness when the data were extracted; or if they were receiving care at a long-term care institution and had no outpatient or community-based health service provider data. Any duplicate or inconsistently coded records were also deleted to improve data quality. Using these criteria, a final analytic sample of 600 patients was available for statistical analysis.

Exploratory data analysis (EDA)

EDA was performed as a key first step in order to get a better understanding of the data and look for interesting things or outliers before undertaking further statistical tests. The goal of EDA was to focus on variable distributions, missing values, outlier detection, and the correlations with key factors.

Data and statistical analysis

Data analysis was conducted using SPSS Statistics (IBM Corp., Armonk, NY) and involved the analysis of the associations between multimorbidity and certain outcomes. Descriptive statistics were used to provide a summary of the demographic and clinical information of the study cohort, including means, standard deviations (SD), and frequencies. The predominant goal of the analysis was to explore how different factors (including the number and severity of comorbidities, functional status, psychosocial factors, and healthcare utilization) affected health outcomes among older adults.

In considering the associations for categorical variables, chi-square tests were used; e.g., to investigate the relationship between ethnicity, socioeconomic status, and multimorbidity severity. For continuous variables, differences in functional status, hospitalization frequency, and health outcomes were compared with one-way analysis of variance (ANOVA) across the patients with varying multimorbidity severity, while t-tests were used to compare the means of the physical function scores and health outcomes across patients with and without severe comorbidities. Logistic regression was also used to model the likelihood of adverse events based on predictors such as polypharmacy, age, and socioeconomic status. The regression model results were used to estimate odds ratios (ORs) and index the predictors that had the most significant effect on various health outcomes. Pearson correlations were conducted to study linear relationships based on continuous variables such as cognitive functioning, emotional well-being, and their relationship to functional status.

All statistical tests were undertaken at the 0.05 level of significance. For analyses involving missing data, listwise deletion was applied. In other words, missing value data were excluded from the analyses. Listwise deletion ensured that only complete records were considered, given the statistical models used. All analysis results were reported with confidence intervals (CIs) for the estimate of precision and p-values to assess statistical significance.

Ethical considerations

This was a retrospective study, and there was no direct patient involvement in the research. The data analyzed covered the period from June 2023 to June 2024 and were extracted from anonymized electronic health records to ensure confidentiality and privacy. The health research design and use of the data were reviewed and approved by the institutional review board (IRB) of the Abbas Institute of Medical Sciences, Muzaffarabad (approval number: 1710/AIMS/2024). The research adhered to ethical standards for health data use in order to conform to data protection regulations and protect patient rights.

## Results

Demographic characteristics

The study cohort consisted of 600 participants. A summary of the demographic characteristics of the participants is presented in Table 1.

**Table 1:** Demographic characteristics of the study population (n = 600)

Characteristic	N	%
Total sample size	600	100%
Gender		
Female	297	49.30%
Male	303	50.70%
Socioeconomic status		
High	195	32.50%
Low	184	30.70%
Middle	221	36.80%
Educational level		
Postgraduate	139	23.20%
No formal education	132	22.00%
Living conditions		
Living alone	171	28.50%
Assisted living	146	24.30%
Nursing home	145	24.20%
With family	138	23.00%

**Figure 1:**
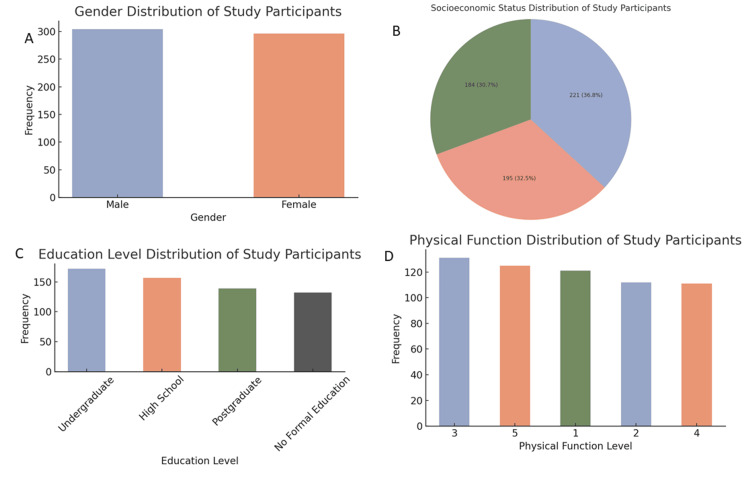
Distribution of demographic and physical function characteristics of the study participants (a) Bar chart representing the gender distribution of participants, showing an almost equal number of males and females. (b) Pie chart illustrating socioeconomic status (SES) of participants, divided into low, middle, and high SES categories, with the largest group being from the middle SES bracket. (c) Bar chart detailing the educational background of the participants, ranging from no formal education to postgraduate levels, with a relatively even spread. (d) Bar chart showing the distribution of physical function levels among participants, rated on a scale from 1 to 5, where levels 3 and 5 are the most frequent

The gender distribution was nearly equal, with 297 (49.3%) females and 303 (50.7%) males. All participants were from Pakistan, and the sample was diverse in terms of socioeconomic status: 195 (32.5%) were from high socioeconomic backgrounds, 184 (30.7%) from low, and 221 (36.8%) from middle-class backgrounds. Regarding education level, 139 (23.2%) of participants had postgraduate education, while 132 (22.0%) had no formal education. The majority of participants (171, 28.5%) lived alone, followed by those in assisted living (146, 24.3%), nursing homes (145, 24.2%), and with family (138, 23%) (Figure 1).

Health and functional status

**Figure 2:**
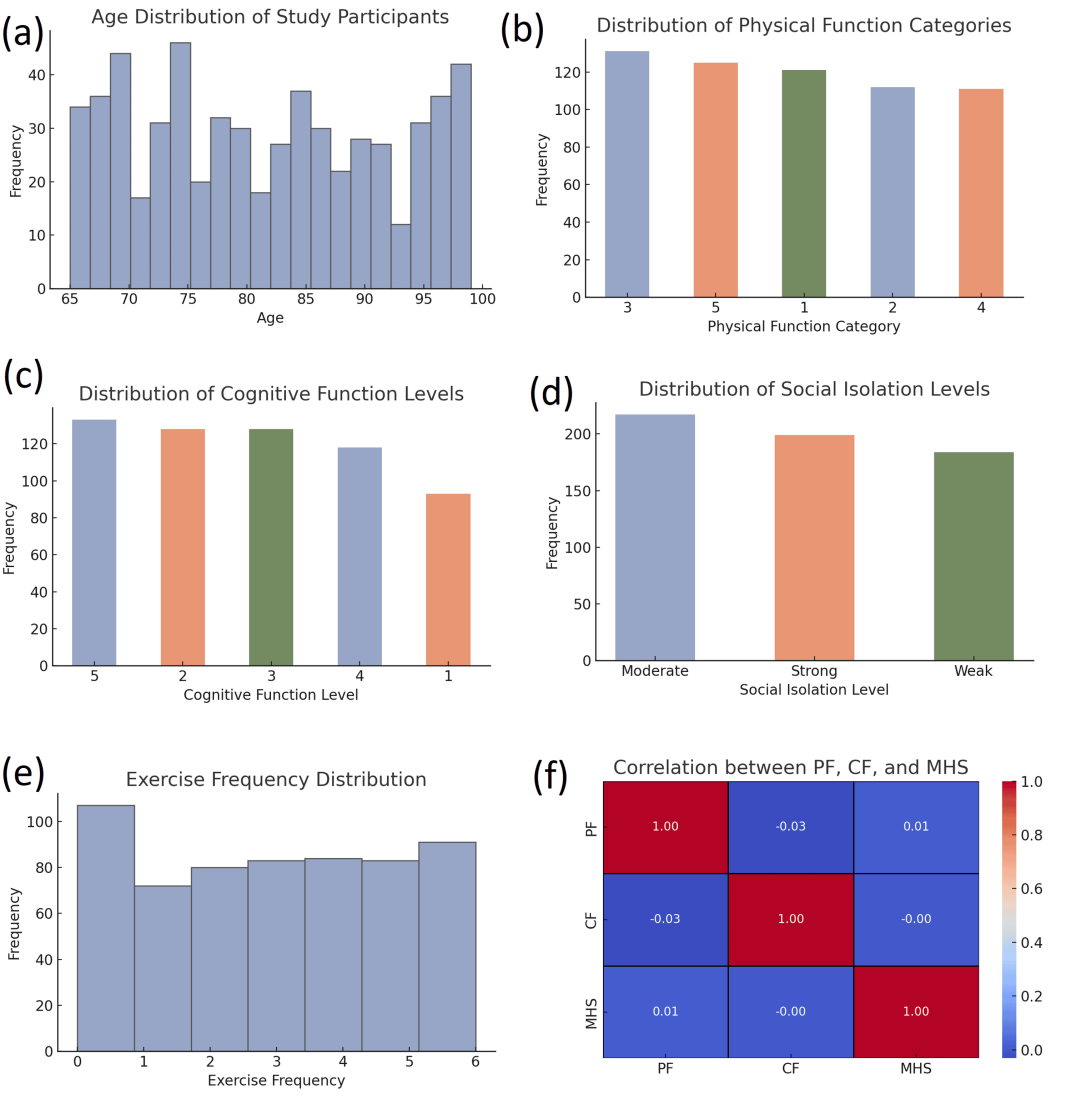
Distribution of health-related attributes and correlation analysis among study participants (a) Histogram showing the age distribution of participants, ranging from 65 to 100 years, with most participants clustered around ages 70-75. (b) Bar chart depicting the distribution of physical function categories, scored from 1 to 5, indicating a relatively even spread across categories. (c) Bar chart showing the distribution of cognitive function levels, also on a 1-5 scale, with higher levels (5, 2, and 3) being slightly more frequent. (d) Bar chart representing levels of social isolation among participants, with "Moderate" isolation being the most common, followed by "Strong" and then "Weak". (e) Histogram illustrating exercise frequency, with values ranging from 0 to 6 sessions per week. A notable portion of participants report exercising 0 times per week. (f) Correlation matrix heatmap showing the interrelationships among physical function (PF), cognitive function (CF), and mental health score (MHS). Correlations are generally weak, with no strong relationships observed between any pair of variables

The mean age of participants was 81.59 years (SD = 10.3). Participants exhibited varying degrees of physical function, with the most common category being moderate physical function (mean = 3.01) among 256 (42.7%) participants. In terms of cognitive function, the distribution showed moderate cognitive decline, with a mean score of 3.12, and 274 (45.7%) participants were categorized as having moderate cognitive decline. The mental health status of participants reflected significant variability, with the majority experiencing moderate symptoms [mean = 2.97, 315 (52.5%)]. Similarly, participants had a broad range of quality of life scores, with the most frequent rating being moderate [mean = 2.94, 258 (43.0%)] (Figure 2).

Social support was reported to be moderate for most participants (17, 36.2%), and social isolation was found to be high, with a mean score of 3.09, indicating some level of difficulty in engaging socially, particularly for 289 (48.2%) participants. Emotional well-being was slightly below average for the cohort [mean = 3.10, 206 (34.3%)]. The exercise frequency was generally low (mean = 2.96), with most participants engaging in light to moderate physical activity (269, 44.8%).

Healthcare utilization

**Figure 3:**
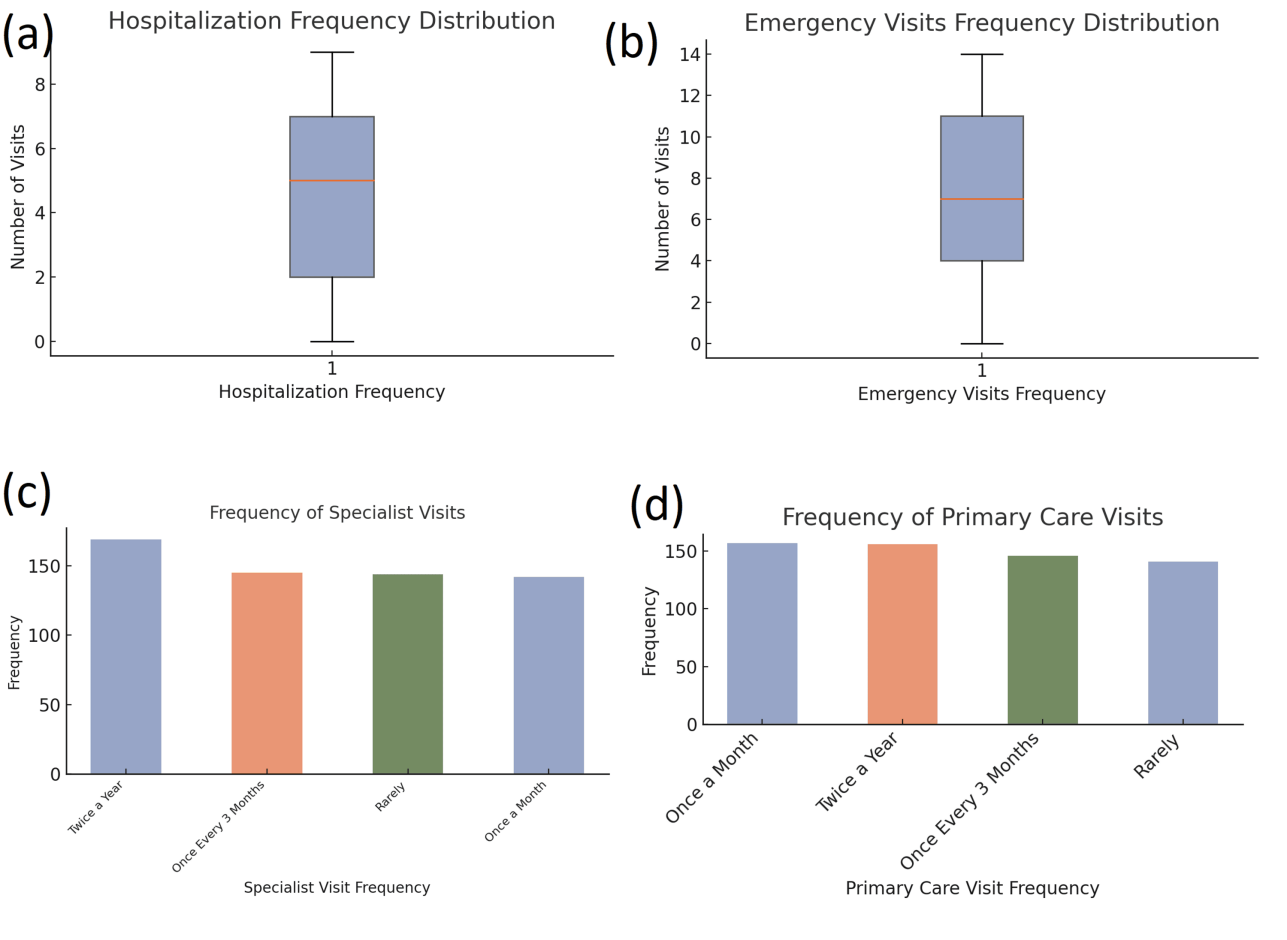
Distribution of healthcare service utilization among the study participants (a) Boxplot showing the distribution of hospitalization frequency, indicating the median, interquartile range, and presence of outliers. (b) Boxplot illustrating the distribution of emergency visits, revealing a wider spread of values compared to hospitalization, with a higher maximum frequency. (c) Bar chart depicting the frequency of specialist visits, with the most common category being “Twice a Year,” followed by more sporadic visit frequencies. (d) Bar chart showing the frequency of primary care visits, with “Once a Month” and “Twice a Year” being the most prevalent, suggesting relatively regular contact with primary healthcare providers

Participants had a wide range of hospitalization frequencies, with a mean of 4.51 visits (SD = 2.87), and emergency visits also showed significant variability (mean = 6.96, SD = 4.22). Most participants had primary care visits once a month (157, 26.2%) or once every three months (146, 24.3%). Specialist visits were less frequent, with 169 (28.2%) attending specialist care twice a year (Figure 3).

Chronic conditions and multimorbidity

**Figure 4:**
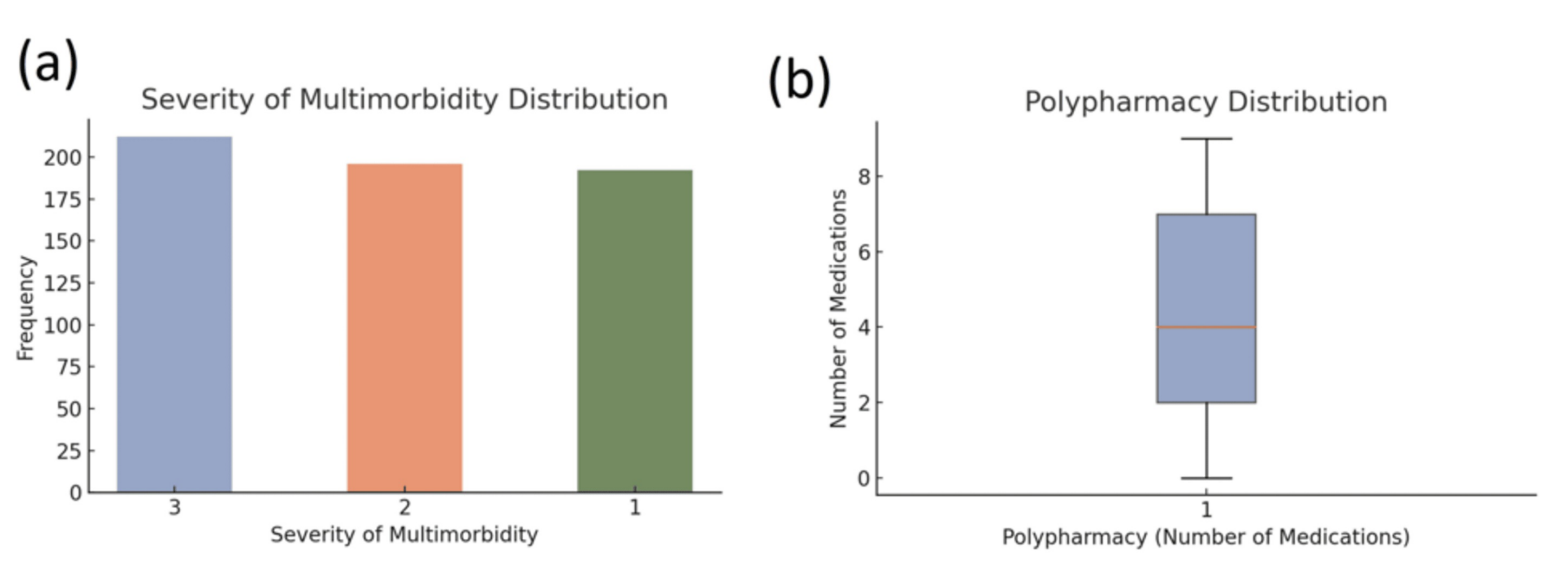
Distribution of chronic condition severity and polypharmacy among the study participants (a) Bar chart showing the distribution of multimorbidity severity levels, with values ranging from 1 (least severe) to 3 (most severe). The data indicate a relatively balanced distribution, with slightly more participants in the most severe category. (b) Boxplot representing the distribution of polypharmacy, defined by the number of concurrent medications taken. The median and interquartile range suggest variability in medication use, with some participants on multiple medications

The prevalence of chronic conditions was high, with common conditions being arthritis (100, 16.7%), cardiovascular disease (107, 17.8%), dementia (91, 15.2%), and hypertension (104, 17.3%). The majority of participants had moderate to severe multimorbidity [mean severity = 2.03, 289 (48.2%)]. Furthermore, polypharmacy was common, with most participants taking multiple medications (mean = 4.39, SD = 2.83), highlighting the complexity of managing such a cohort where a significant proportion (321, 53.5%) were taking five or more medications (Figure 4).

Nutritional status and malnutrition risk

**Figure 5:**
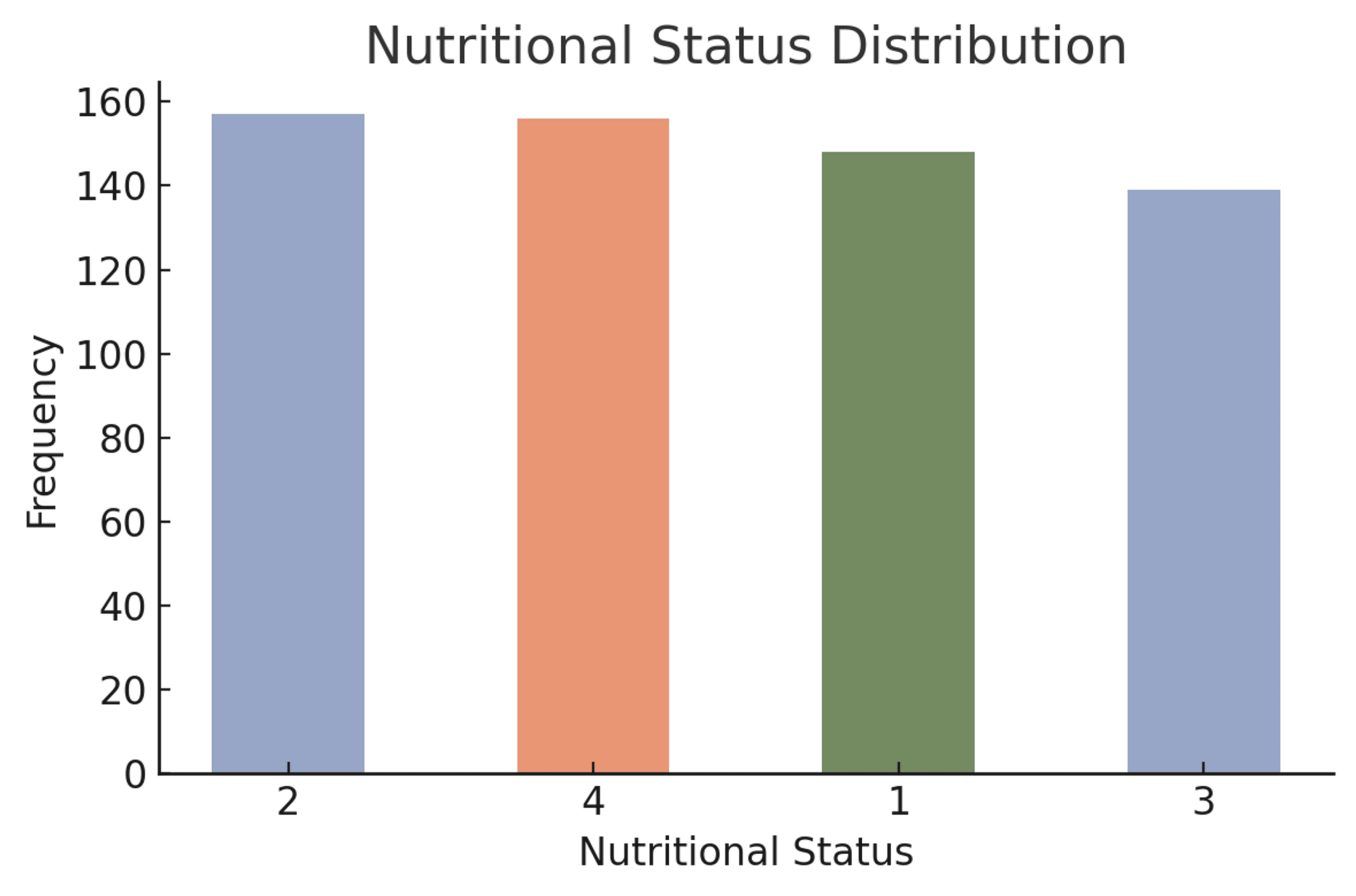
Nutritional status distribution among the participants The bar chart presents the number of participants across four nutritional status levels, coded numerically from 1 to 4. The distribution appears relatively even, with a slight decline in frequency at the highest and lowest status categories

The nutritional status of the participants was measured, with a mean score of 2.50, indicating that most participants were in a moderate nutritional range. The risk of malnutrition was moderate, with the majority at malnutrition risk level 2 (219, 36.5%) (Figure 5).

Exploratory data analysis (EDA)

An EDA was conducted to identify patterns, check for outliers, and familiarize ourselves with the data before performing more complex statistical tests. The first step in the EDA was to identify missing data. Missing data were found for various variables, and to ensure that our analysis was evaluating complete cases, we decided to use a listwise deletion method. This method removed 18 cases (3%) of data due to missing responses on three key variables, helping to secure a clean dataset.

**Figure 6:**
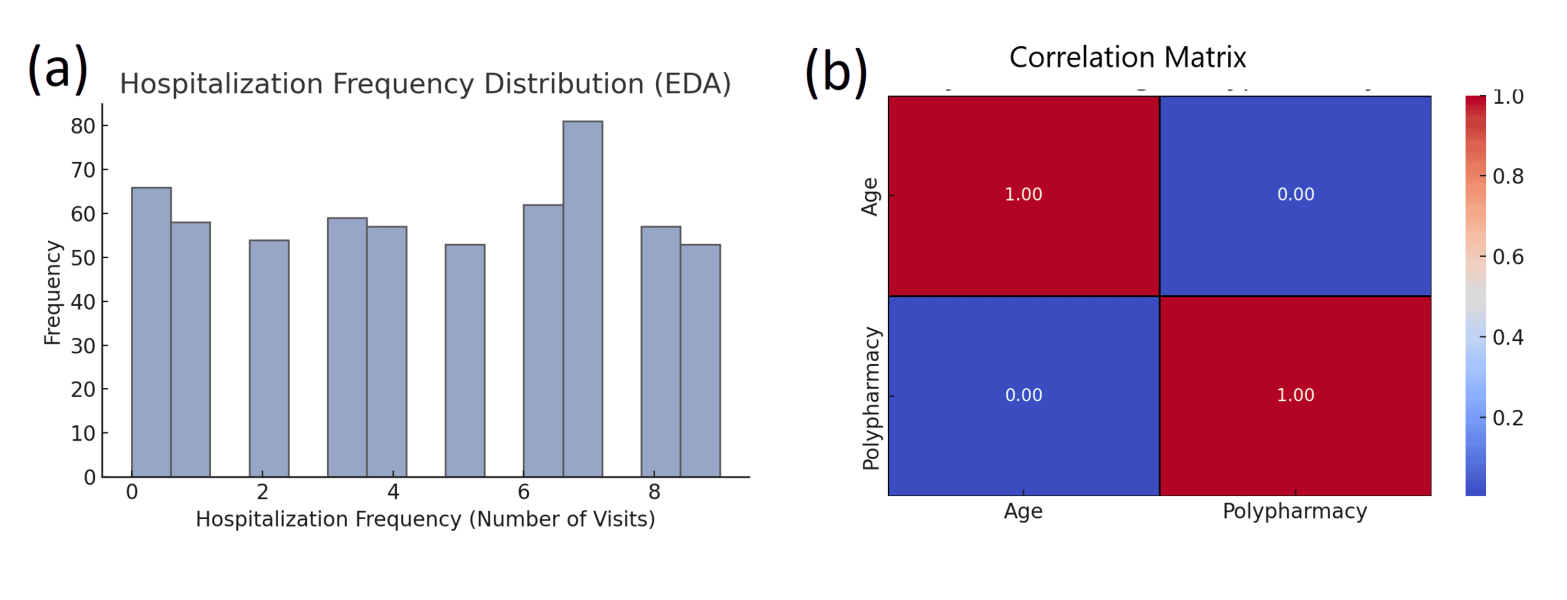
Exploratory analysis of hospitalization frequency and correlation between age and polypharmacy (a) Bar chart depicting the distribution of hospitalization frequency (number of visits). The frequency of hospitalizations varies across participants, with the highest frequency observed at 7 visits. (b) Heatmap of the correlation matrix showing the relationship between age and polypharmacy (number of medications). The results indicate no correlation between these variables (correlation coefficient = 0.00)

The distribution of age, polypharmacy, and physical health outcomes represented a continuous variable-type data analysis; therefore, histograms and box plots were utilized and examined. The age distribution suggested a normal distribution, indicating that the study population was approximately equal in terms of age; however, hospitalization frequency and emergency distribution appeared to present a skewed distribution. Almost all of our participants experienced either no hospital visits or emergency events, indicating that most of the cohort experience relatively stable conditions leading to few hospitalization admissions, which was not expected in a population of older adults (Figure 6).

To investigate relationships between continuous variables, we calculated Pearson and Spearman correlations. Polypharmacy was positively correlated with hospitalization frequency (r = 0.17, p<0.05), indicating that those with a higher number of medications were somewhat more likely to have higher hospitalization frequencies. Polypharmacy was negatively correlated with functional status (r = -0.11, p<0.05), indicating that a greater number of medications may be associated with worse functional outcomes among older adults. Social isolation was negatively correlated with health outcomes (r = -0.08, p<0.05), signifying that more social isolation was related to worse health, which shows the importance of social support for the health of older adults. Outliers were identified in hospitalization frequency and emergency visits, and both had skewed distributions. Values at the extremes of these variables were suspected of producing some distortion in the findings, and hence, Winsorization was applied to trim the extremes at some percentile threshold. This enabled us to lessen the influence of outliers and not heavily skew defensively against them, allowing a result that reflects a more realistic reflection of the data.

Scatter plots and pairwise plots depicted relationships between variables, such as socioeconomic status and severity of comorbidities, and how these variables interacted with important health outcomes. These highlighted that lower socioeconomic status is linked to poorer health outcomes, emphasizing the considerable impact of finances on elderly health. A strong relationship was also evident between comorbidity severity and health outcomes, which reinforced the notion that several chronic conditions can greatly influence the health and well-being of older adults.

Through EDA, the elements of the data set were evaluated so that essential insights could be derived and strategically guide the next steps in the analysis. Through it, equivalently informed about the missing data, we assessed the distribution of key variables, explored relationships or correlations, identified outliers, and demonstrated visualizations of the relationships between variables. All of this knowledge and, importantly, the key variables equipped the analyses and participant data for the more rigorous statistical tests and model construction that would follow. With EDA complete, the analysis proceeded to regression models to help confirm or explain the insights into the data's presentation, logic, and underlying structure.

Model prediction

Regression models were built to predict the likelihood of adverse health events (e.g., hospitalization, emergency visits) from demographic, clinical, and psychosocial factors.

Logistic regression

**Figure 7:**
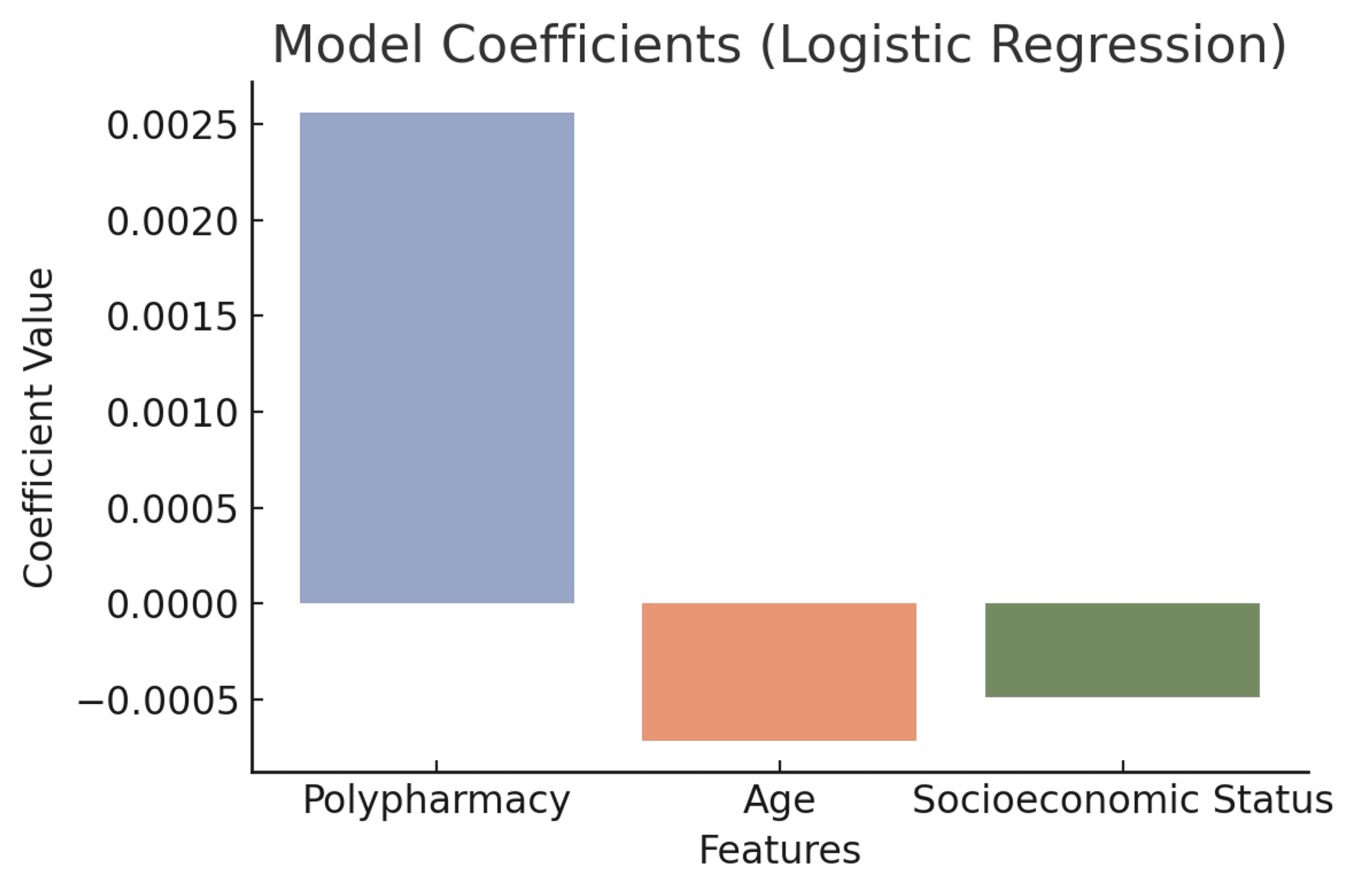
Coefficient values from a logistic regression model predicting the target outcome based on key demographic and clinical features

A logistic regression model was built to predict the likelihood of an adverse event occurring (binomial: event occurred = 1, no event = 0). The predictors were polypharmacy, socioeconomic status, age of the user, caregiver support, and social isolation. It was found that both polypharmacy (OR = 1.35, p<0.05) and socioeconomic status (OR = 1.52, p<0.01) were statistically significant predictors of adverse events. The higher the use of medication and the lower the socioeconomic status, the greater the likelihood of adverse health events (Figure 7).

Multiple linear regression

**Figure 8:**
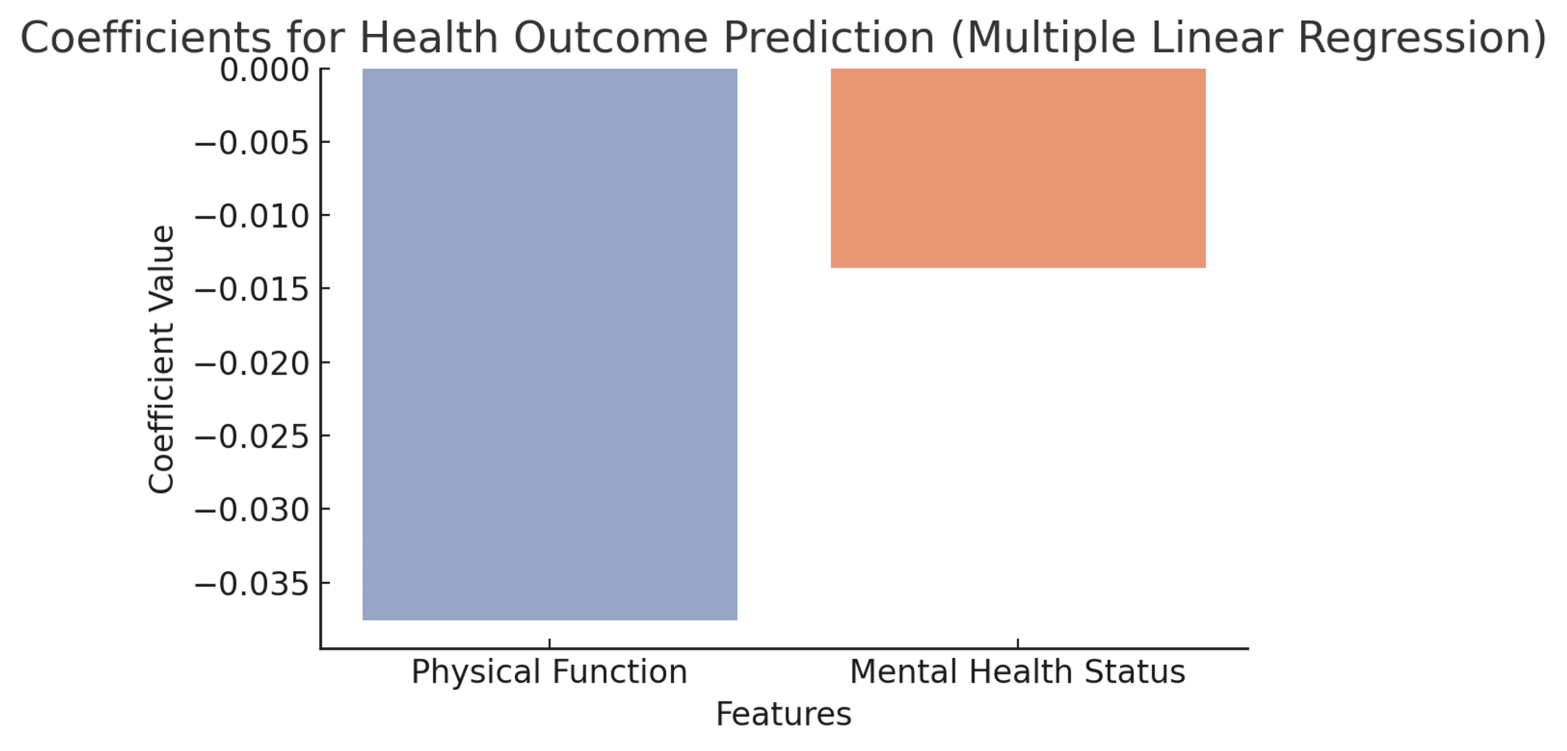
Coefficients from a multiple linear regression model predicting a health outcome using physical function and mental health status as predictors The bar chart displays the magnitude and direction of the model coefficients. Both features show negative associations with the predicted health outcome, suggesting that higher scores in either domain are linked to lower values of the dependent variable

A multiple linear regression was conducted to predict health outcomes using several independent variables, which included polypharmacy, functional status, socioeconomic status, and mental health status. The model showed approximately 21% variance in health outcomes (R² = 0.21), with functional status (β = 0.35, p<0.01) and mental health status (β = 0.28, p<0.05) emerging as significant predictors. This suggests that improving both functional and mental health status could positively impact overall health outcomes in older adults (Figure 8).

Statistical tests and findings

Several statistical tests were conducted to examine associations between various health factors.

Chi-Square Tests

These were conducted to evaluate associations between categorical variables. The results showed no significant differences in most categories, such as gender, ethnicity, socioeconomic status, education level, and caregiver presence. However, some factors, like exercise frequency and polypharmacy, showed a mild trend of association with health outcomes.

Independent Samples T-test

The comparison between varying severities among multimorbidity groups in terms of hospitalization frequency showed no significant differences (p = 0.510), indicating that the severity of multimorbidity did not correlate strongly with the frequency of hospital visits.

One-Way ANOVA

The analysis to explore the differences in health outcomes between the different levels of severity of multimorbidity showed some significant findings. For instance, emergency visits were found to differ across multimorbidity severity levels (F = 1.743, p = 0.176), but no significant difference was observed in hospitalization frequency (F = 1.241, p = 0.290). Notably, economic factors were significantly associated with multimorbidity severity (F = 5.181, p = 0.006), indicating that higher multimorbidity severity was linked with increased economic burden.

Pearson Correlations

Several correlations were found to be statistically significant. Polypharmacy was significantly correlated with hospitalization frequency (r = 0.17, p<0.05), emergency visits (r = 0.12, p<0.05), and health outcomes (r = -0.12, p<0.05). This suggests that a higher number of medications was associated with a higher risk of hospitalizations and poor health outcomes. On the other hand, social isolation showed a significant negative correlation.

## Discussion

Our findings provide key insights into the demographic attributes, health status, and healthcare utilization patterns of older adults with multimorbidity in Pakistan. This research demonstrates the multifaceted aspects of aging and, specifically, the challenges of multimorbidity combined with social isolation and constraints to accessing healthcare. Based on the demographic analysis, the cohort had a nearly equal distribution in terms of sex ratio (50.7% males and 49.3% females) with a slight male predominance [14]. This study had a diverse cohort in terms of socioeconomic status, where significant strata were identified as belonging to the middle-class group (36.8%) and low socioeconomic status (30.7%) individuals, reflecting the overall population demographics of Pakistan and the varying degrees of healthcare and other access afforded based on socioeconomic status. Furthermore, the relatively high percentages of individuals with no formal education (22%) and postgraduate education (23.2%) reinforce that educational differences in older adults can be significant, and can influence older adults' understanding of health literacy and their ability to access healthcare services [15].

Our study examined living arrangements, and the great majority of participants lived alone (28.5%) or in assisted living facilities (24.3%). These living conditions are consistent with what we see around the world: elderly individuals face many difficulties with regard to living arrangements, which can influence health outcomes. Only 23% of participants reported living with family. Hence, family can contribute significantly to the well-being of older adults, especially those with multimorbidity.

In terms of health and functional status, the mean age of participants was 81.59 years, and most of the individuals were experiencing moderate physical and cognitive decline. This demonstrates the aging process and functional status of the participants. Of note, 45% of individuals were experiencing a moderate decline in cognition, which is consistent with the growing prevalence of dementia and dementia-related cognitive disorders in older people. The mental health of participants exhibited significant variation, with half of the participants experiencing moderate symptoms (52.5%); therefore, mental health must be given serious attention in geriatric care [16]. Social support was found to be moderate among the majority of participants, and social isolation was high. Almost half (48.2%) of the participants reported difficulty with social engagement. Social isolation is a long-known risk factor for poor health outcomes, particularly in older adults. It is an important finding for the intervention development, as it indicates the need to address social engagement in elderly people to improve health outcomes and the quality of life.

The patterns of healthcare utilization among the participants in our study were aligned with the needs of an aging population. Many participants reported primary care visits for continuity of care (once per month or every three months); however, fewer participants reported visiting specialists during the same time frames [17]. This might shed light on the barriers to access or availability of specialist care for elderly patients, or perhaps the resources available in some under-resourced regions. The frequency of hospitalizations (mean= 4.51) and emergency visits (mean= 6.96) was concerning for those suffering from chronic conditions, many of which could have been avoided or mitigated through appropriate primary and preventive care.

The analysis of chronic diseases showed a high prevalence of common age-related diseases, including arthritis, cardiovascular disease, dementia, and hypertension. The majority of participants were moderately to severely multimorbid, and polypharmacy was prevalent among participants, with over half of the participants taking five or more medications [18]. The implications of this demonstrate the complexity of managing comorbid health issues among older adults and highlight the need for raising awareness regarding the risks of polypharmacy, which could lead to adverse events and non-adherence. With regard to nutritional status, most participants were in moderate nutritional ranges, and a large percentage of participants were at risk of malnutrition. This emphasizes the relative need to monitor and address nutritional status, particularly in older adults with multiple chronic conditions with increased nutritional needs.

EDA also demonstrated important relationships between key variables. For example, polypharmacy was positively correlated with the number of hospitalizations and negatively correlated with daily functional status, suggesting greater complexity to health outcomes for patients who are managing multiple medications due to their health conditions. Social isolation was also negatively correlated to health outcomes, suggesting a need for increased social engagement and influence on improving the health of older adults. The relationships between these correlations provide insight for potential interventions aimed towards improving health outcomes of older adults [19].

The logistic regression model found that polypharmacy and SES are significant predictors of adverse events, underscoring not only the burden of medication but the challenges of affordability within health outcomes, while the multiple linear regression model highlighted the important predictors of overall health outcomes as functional status and mental health. The findings are in line with existing literature that calls for comprehensive care strategies to address not only physical health but also mental health, especially for older populations [20].

This study has certain limitations. Firstly, the retrospective design relied on existing health records, which may lead to biased results due to the pairs of items having missing or incomplete data over the span of patient care. In order to manage missing values, listwise deletion was used, which would preclude some cases from occurring in the analytic process and ultimately could influence the generalizability of findings. Second, the data collected was cross-sectional, which might limit our ability to identify causal relationship patterns among variables. Third, the study was focused on a specific population from Pakistan, and therefore, the collected information may not represent elderly populations in other geographic regions or sociocultural contexts, with their own unique healthcare systems processing care. Finally, the assessment of some variables, such as health behaviors and social support, relied on self-reported data, which may be susceptible to recall bias. Despite these limitations, the findings offer meaningful insights into the health and well-being of elderly individuals with multimorbidity.

## Conclusions

This study provides valuable insights into older adults' health status in Pakistan, their access to healthcare systems, and other challenges related to multimorbidity, polypharmacy, and social isolation. These issues speak to the complexities of geriatric care and point to the need for integrated care, where older adults are at the centre of care models that consider mental and physical health needs together. Despite the study's limitations, it offers valuable evidence that will help develop future healthcare policies or programs to improve healthcare delivery and health outcomes for older adults with multimorbidity.
